# Case Report and Review of Literature: Autosomal Recessive Hypophosphatemic Rickets Type 2 Caused by a Pathogenic Variant in *ENPP1* Gene

**DOI:** 10.3389/fendo.2022.911672

**Published:** 2022-07-29

**Authors:** Yunsoo Choe, Choong Ho Shin, Young Ah Lee, Man Jin Kim, Yun Jeong Lee

**Affiliations:** ^1^ Department of Pediatrics, Seoul National University Children’s Hospital, Seoul, South Korea; ^2^ Department of Genomic Medicine, Seoul National University Hospital, Seoul National University College of Medicine, Seoul, South Korea

**Keywords:** rickets, autosomal recessive hypophosphatemic rickets, ectonucleotide pyrophosphatase phosphodiesterase 1, child, case report

## Abstract

Autosomal recessive hypophosphatemic rickets type 2 (ARHR2) is a rare form of hereditary rickets, which is characterized by defective bone mineralization and renal phosphate wasting due to a loss-of-function variant in the ectonucleotide pyrophosphatase/phosphodiesterase 1 (*ENPP1*) gene. Although pathogenic variant of *ENPP1* has been known to manifest other phenotypes including arterial calcification, hearing loss, ossification of posterior longitudinal ligament, or pseudoxanthoma elasticum, there have been few reports including systematic examination in individuals diagnosed with ARHR2 to date. Herein, we report a case of ARHR2 with a bi-allelic pathogenic variant of *ENPP1*, in which the patient presented with gait abnormalities with severe genu varum at 26 months of age. Targeted gene panel sequencing was performed to investigate the genetic cause of rickets, and a homozygous nonsense variant in *ENPP1*, c.783C>G (p.Tyr261*), was identified. The patient was treated with oral phosphate and active vitamin D supplements and underwent corrective osteotomy for varus deformity. His phenotype was limited to rickets. A periodic systematic evaluation is needed to identify any comorbidities in ARHR2 patients since *ENPP1* variants may present phenotypes other than rickets and symptoms may evolve or change over time.

## Introduction

Autosomal recessive hypophosphatemic rickets type 2 (ARHR2; OMIM 613312) is a rare form of hereditary hypophosphatemic rickets, characterized by bone mineralization defects due to renal phosphate wasting (1). ARHR2 is caused by bi-allelic pathogenic variants in the *ENPP1* gene, which encodes ectonucleotide pyrophosphatase/phosphodiesterase 1 (ENPP1), a major generator of extracellular inorganic pyrophosphate (PPi) and an inhibitor of fibroblast growth factor 23 (FGF23) (2). The inactivating variants of *ENPP1* also cause ectopic calcification and over-mineralization in arteries, ligaments, and skin matrix, leading to various allelic disorders such as generalized arterial calcification of infancy (GACI), early-onset hearing loss, ossification of posterior longitudinal ligament (OPLL), and pseudoxanthoma elasticum (PXE) (3–6). To date, a few cases of ARHR2 have been reported but no studies have investigated accompanied phenotypes comprehensively. Here, we report a rare case of ARHR2, evaluating other *ENPP1*-related disorders through systematic examination. And we also review the genotypes and phenotypes of previously reported ARHR2 patients.

## Case Presentation

**Figure 1:**
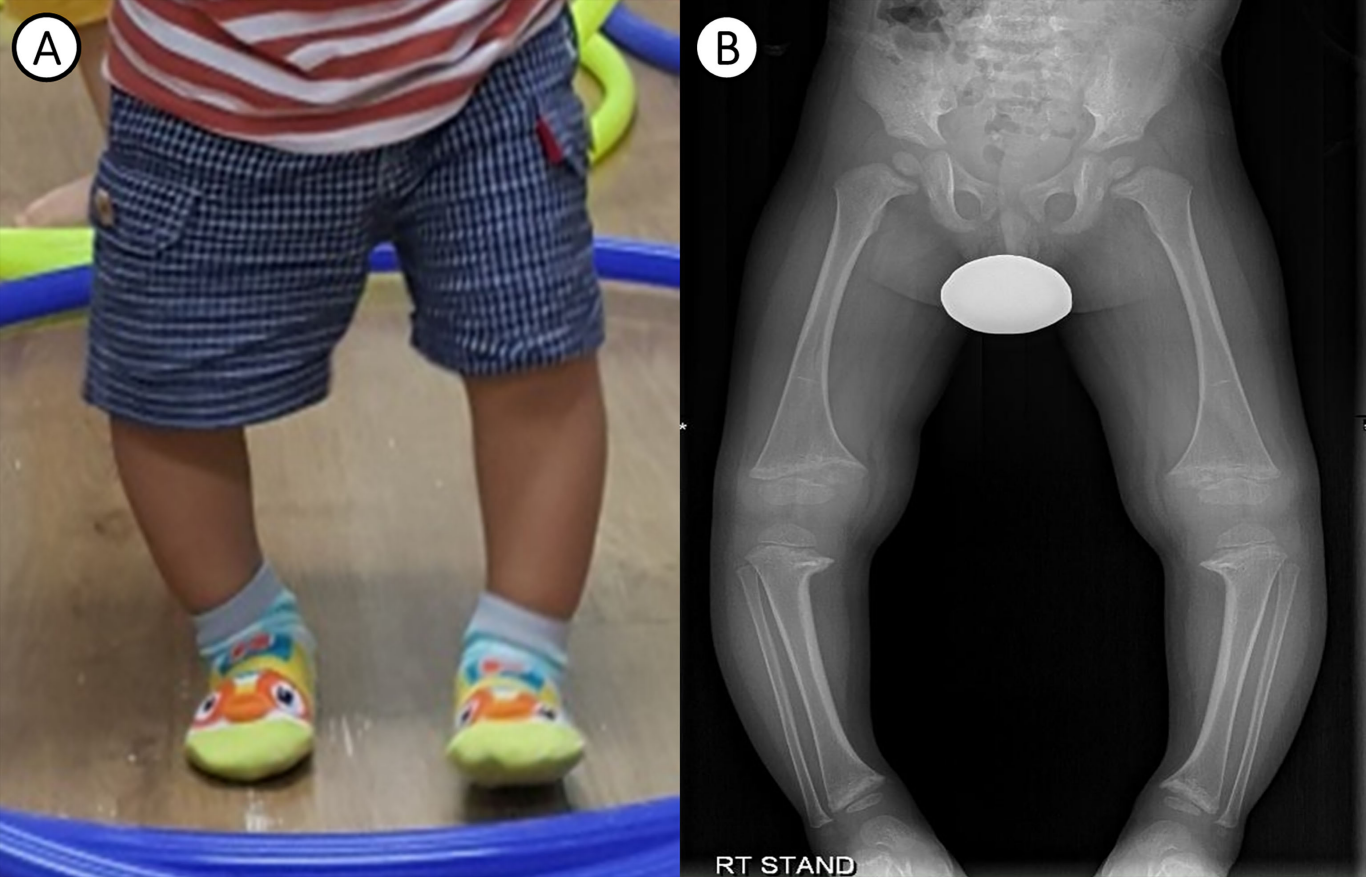
Clinical photograph and radiological findings of the patient. **(A)** Photograph of the patient’s bowed legs at 24 months of age. **(B)** Plain radiograph of the lower extremities showing bilateral metaphyseal flaring of the distal femur and tibia, associated with varus deformity, at 30 months of age.

A 26-month-old male was referred to a tertiary care center for an evaluation of genu varum in October 11, 2017. He was delivered by cesarean section at 38 weeks of gestation, with a weight of 3.2 kg, to non-consanguineous Korean parents. No abnormalities were detected in prenatal screening test and the patient didn’t have intrauterine growth retardation (IUGR). He had no cardiovascular problems in infancy and had age-appropriate psychomotor development. He exhibited bowed legs at 12 months, after he started walking. At presentation, he had a short stature, with a height of 79.3 cm (-2.99 standard deviation score [SDS]) and a weight of 11.4 kg (-0.83 SDS). Physical examination revealed significant varus deformities of the bilateral lower extremities (**Figure 1A**) and a waddling gait pattern. Craniotabes or rachitic rosary were not observed. Laboratory examinations (**Table 1**) revealed low serum phosphorus (2.6 mg/dL, reference range 4.1-6.2 mg/dL), elevated serum alkaline phosphatase (ALP) (472 IU/L, reference range 39-117 IU/L), and normal serum calcium (9.5 mg/dL, reference range 8.0-10.5 mg/dL). 25-hydroxyvitamin D (32.9 ng/mL, reference range 30-100 ng/mL) and intact parathyroid hormone (40.55 pg/mL, reference range 15.0-65.0 pg/mL) levels were in the normal range. He showed renal phosphate wasting, with decreased tubular resorption of phosphate (TRP; 83.3%, reference range ≥ 85%) and decreased tubular maximum reabsorption of phosphate per unit volume of glomerular filtration rate (TmP/GFR; 2.16 mg/dL, reference range 2.9~6.5 mg/dL). He had no hypercalciuria. X-ray of the lower extremities revealed bilateral tibia vara with metaphyseal beaking and flaring of the proximal tibia, distal tibia, and distal femur (**Figure 1B**). He was prescribed 250 mg of oral phosphate salts (21.9 mg/kg/day) and 0.25 mcg of calcitriol (0.02 mcg/kg/day).

**Table 1:** Clinical characteristics and laboratory findings of the patient with ARHR2 and his family.

Subject	ll-2	l-1	l-2	ll-1
Relationship	Proband	Father	Mother	Sister
Age at initial presentation, years	2	–	–	–
Age at genetic diagnosis, years	5	47	43	9
Sex	Male	Male	Female	Female
Present height, cm [SDS]	105.8 [-2.25]	173 [-0.24]	154 [-1.48]	126.8 [-1.09]
Present weight, kg [SDS]	17.0 [-1.93]	82	69	23.9 [-1.42]
Variants in *ENPP1* gene	c.783C>G (p.Tyr261*) homozygous variant	c.783C>G (p.Tyr261*) heterozygous variant	c.783C>G (p.Tyr261*) heterozygous variant	c.783C>G (p.Tyr261*) heterozygous variant
*Clinical characteristics*				
Bowing deformity	+	–	–	–
Hearing loss	–	–	–	–
History of vascular calcification in infancy	–	–	–	–
Bone pain	–	–	+	–
Fracture	–	–	–	–
Neurological symptoms	–	–	–	–
Bone mineral density	n.c	Normal	Normal	n.c
Spinal ligament ossification	–	–	–	–
Blood pressure	Normal	Prehypertension	Hypertension	Normal
*Laboratory and image findings*				
Calcium, mg/dL	9.5 (8.0-10.5)	8.9 (8.8-10.5)	9.2 (8.8-10.5)	9.7 (8.8-10.5)
Phosphorus, mg/dL	2.6 (4.1-6.2)	3.3 (2.5-4.5)	2.9 (2.5-4.5)	5.3 (3.6-5.8)
Alkaline phosphatase, IU/L	472 (39-117)	129 (30-115)	95 (30-115)	200 (154-391)
Intact parathyroid hormone, pg/mL	40.55 (15-65)	28 (8-76)	66 (8-76)	9 (8-76)
25-hydroxyvitamin D, ng/mL	32.9 (30-100)	14.6 (30-100)	10.2 (30-100)	12.9 (30-100)
1,25-dihydroxyvitamin D, pg/mL	26.25 (18.7-47.7)	41.3 (19.6-54.3)	41.9 (19.6-54.3)	30.1 (19.6-54.3)
Urine calcium/creatinine ratio	0.049 (< 0.2)	0.01 (< 0.2)	0.19 (< 0.2)	0.05 (< 0.2)
Tubular resorption of phosphate, %	83.3 (≥ 85)	87 (≥ 85)	83 (≥ 85)	94 (≥ 85)
TmP/GFR, mg/dL	2.16 (2.9-6.5)	n.c	n.c	n.c
Carotid intima-media thickness (cIMT)	Increased	n.c	n.c	n.c
Echocardiography	No aortic calcification	n.c	n.c	n.c

Reference values are presented in parenthesis next to the individual value for age-specific criteria.ARHR2, autosomal recessive hypophosphatemic rickets type 2; SDS, standard deviation score; TmP/GFR, tubular maximum reabsorption of phosphate per unit volume of glomerular filtration rate; n.c, not conducted.

**Figure 2:**
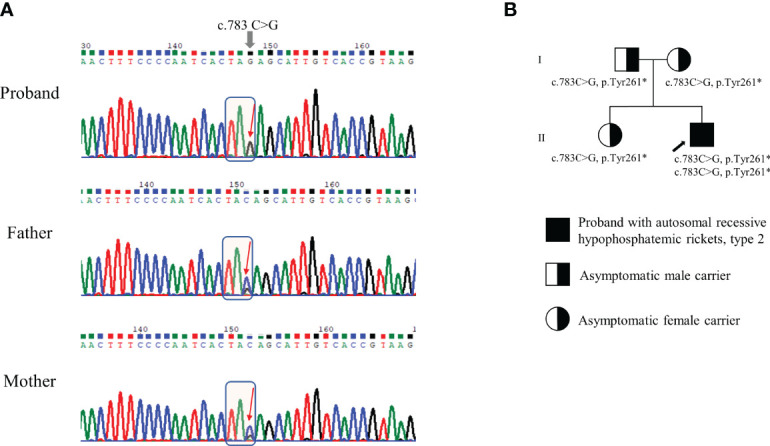
Genetic analysis of *ENPP1* gene and pedigree of the family. **(A)** Genomic DNA sequences of the patient and his parents exhibiting either homozygous or heterozygous c.783C>G (p.Tyr261*) variant in the *ENPP1* gene. **(B)** Pedigree of the family. The variants identified in the *ENPP1* gene are indicated below each individual. The black arrow indicates the proband.

We conducted Sanger sequencing to analyze genes commonly associated with hypophosphatemic rickets, including *PHEX*, *FGF23*, and *DMP1*. No variants were observed. Further genetic analysis *via* next-generation sequencing (NGS) was conducted using a panel of 21 genes associated with rickets or calcium/phosphate metabolism disorders (including *ENPP1*, *FAM20C*, *SLC34A3*, and *VDR*; **Supplementary Table 1**). The detailed exome sequencing procedure is described in the Supplementary Methods. The detected sequence variants were confirmed by Sanger sequencing analysis using custom-designed primers. We identified a homozygous nonsense pathogenic variant in exon 7 of the *ENPP1* gene, c.783C>G (p.Tyr261*) (**Figure 2A**). Segregation analysis showed that his parents and sister were heterozygous from the same variants (**Figure 2B**). The mother had a short stature (154 cm, -1.48 SDS). She did not complain of bone pain other than her legs, nor did she have any neurological symptoms. (**Table 1**). Her laboratory results showed low TRP (83%) and low 25-hydroxyvitamin D (10.2 ng/mL). Dual energy X-ray absorptiometry indicated a normal bone mineral density, with no evidence of OPLL at spinal x-rays. She had taken antihypertensive drugs for 5 years. Both the patient’s father and sister had a normal stature, and there were no bone pain or neurological symptoms. Skeletal X-ray evaluations of the spine and extremities showed no ectopic calcification or OPLL. They had no laboratory abnormalities except for vitamin D deficiency (**Table 1**). None of the family members had a history of fracture.

Despite good compliance with oral medication, the leg deformities did not improve completely, and the patient underwent corrective osteotomy for genu varum at the age of 3 years. At the age of 6, he achieved partial catch-up growth, with a height of 105.8 cm (-2.25 SDS) and a weight of 17.0 kg (-1.93 SDS). His varus deformity was corrected, with an intercondylar distance of 1 cm. Oral medication was maintained at 250 mg of phosphate five times a day (73.5 mg/kg/day), 0.5 mcg of calcitriol (0.03 mcg/kg/day), and 1,000 IU/day of cholecalciferol. Kidney ultrasonography revealed no evidence of nephrocalcinosis. As loss-of-function variants in *ENPP1* gene produce various phenotypes, a cardiovascular evaluation (echocardiography, carotid intima media thickness), auditory evaluation, spinal X-ray, and ophthalmologic examination were performed. No evidence of vascular calcification, hearing loss, OPLL, or PXE were observed.

## Discussion

We describe a rare case of ARHR2 with a bi-allelic *ENPP1* pathogenic variant causing hypophosphatemic rickets. The patient did not exhibit arterial calcification, hearing loss, OPLL, or PXE, which were also been associated with this variant.

ARHR2 is a rare form of hereditary rickets caused by loss-of-function variants in *ENPP1*. Less than 20 cases have been reported worldwide (**Table 2**) (1, 4, 6–12). *ENPP1* comprises 25 exons (located at 6q23) and encodes ENPP1, which inhibits the production of FGF23, a phosphaturic hormone that decreases phosphate reabsorption by down-regulating the expression of sodium-phosphate cotransporter in the renal proximal tubule and reduces 1α-hydroxylase activity and 1,25-dihydroxyvitamin D synthesis (14). Enhanced FGF23-mediated renal phosphate loss reduces bone mineralization, resulting in hypophosphatemic rickets. Thus far, a total of 140 pathogenic variants of *ENPP1* have been reported in the previous peer-reviewed literature (15, 16), and the Human Gene Mutation Database (HGMD Professional; http://www.hgmd.cf.ac.uk/ac/index.ph, access date: September 13, 2021). Among them, 42 variants are associated with rickets. Clinical characteristics and genotypes of previously reported ARHR2 cases are summarized in **Table 2**. The c.783C>G nonsense variant observed in this case was previously reported in a Chinese girl who presented with a similar phenotype (Case 2, **Table 2**) (7).

**Table 2:** Clinical characteristics and genotypes of ARHR2 patients.

	Sex	Age atinitial presentation^a^ (years)	Age atgenetic diagnosis (years)	Variant 1	Variant 2	Short stature	Height (cm) [SDS]	Leg bowing	Arterial calcification	Hearing loss	OPLL	PXE	Ethnicity	References
1	M	2	5	c.783C>G(p.Tyr261*)	c.783C>G(p.Tyr261*)	+	105.8 [-2.3]	+	–	–	–	–	Korean	This case
2	F	4	11	c.783C>G(p.Tyr261*)	c.783C>G(p.Tyr261*)			+					Chinese	Liu et al. (7)
3	F	10	62	IVS21+1_3 (GTA>CACC)	IVS21+1_3 (GTA>CACC)	+	123	+	–		+		Japanese	Saito et al. (4)
4	F	20s	54	c.323G>T(p.Cys108Phe)	c.1441C>T(p.Arg481Trp)	–	163	+	+^b^	+			Caucasian	Kotwal et al. (8)
5	F	17	53	c.323G>T(p.Cys108Phe)	c.1441C>T(p.Arg481Trp)	+	153	+	+^b^	+^c^	+		Caucasian	Kotwal et al. (8)
6	M	10	19	c.A2722C(p.Tyr901Ser)	c.A2722C(p.Tyr901Ser)	+	152	+	–				Bedouin	Levy-Litan et al. (9)
7	M	9	16	c.A2722C(p.Tyr901Ser)	c.A2722C(p.Tyr901Ser)	+	143	+	–				Bedouin	Levy-Litan et al. (9)
8	M	30	30	c.A2722C(p.Tyr901Ser)	c.A2722C(p.Tyr901Ser)	–	165	–	–				Bedouin	Levy-Litan et al. (9)
9	M	8	17	c.2445-798_2778*867del	c.2445-798_2778*867del	–	167.3 [-1.2]	+	–				Turkish	Lorenz-Depiereux et al. (1)
10	M	2.5	11	c.2445-798_2778*867del	c.2445-798_2778*867del	+	130 [-2.3]	+	–				Turkish	Lorenz-Depiereux et al. (1)
11	M	16.8	20	c.797G>T(p.Gly266Val)	c.797G>T(p.Gly266Val)	+	159.6 [-2.5]	+	–				Turkish	Lorenz-Depiereux et al. (1)
12	M	6	21	c.2248_2249insA(p.Ser750LysfsX6)	c.2248_2249insA(p.Ser750LysfsX6)	+	148 [-5]		–				Israeli Arabic	Lorenz-Depiereux et al. (1)
13	M	5.5	35	c.797G>T(p.Gly266Val)	c.797G>T(p.Gly266Val)	+	151.6 [-3.6]	+	–				Turkish	Lorenz-Depiereux et al. (1)
14	M	1	10	c.797G>T(p.Gly266Val)	c.797G>T(p.Gly266Val)	+	112.5 [-4]		+	+			Turkish	Lorenz-Depiereux et al. (1)
15	F	1.1	42	c.956C>G(p.Thr319Arg)	c.2344C>T(p.Arg782*)	+		+			+^d^		Greek	Mehta et al. (10)
16	M	10	16	c.275G>A(p.Gly92Asp)	c.2230+1G>A	+	163	+	+^e^	+			Austrian	Steichen-Gersdorf et al. (6)
17	F	3	13	c.2026C>T(p.Gln676*)	c.2375A>G(p.Asn792Ser), c.655G>A(p.Gly219Arg)	+	143.6	+	–	+				Steichen-Gersdorf et al. (6)
18	F	4	4	IVS22+1G>A	IVS22+1G>A	+		+						Capelli et al. (11)
19	F			c.755A>G(p.Tyr252Cys)	c.755A>G(p.Tyr252Cys)		–							Oheim et al. (12)
20	M	2.5	14.5	c.755A>G(p.Tyr252Cys)	c.755A>G(p.Tyr252Cys)	+	144.6[-3.5]	+	–	+				Oheim et al. (12), Ferreira et al. (13)

ARHR2, autosomal recessive hypophosphatemic rickets 2; M, male; F, female; SDS, standard deviation score; OPLL, ossification of posterior longitudinal ligament; PXE, pseudoxanthoma elasticum.

a Age when hypophosphatemia, rickets, or bone/joint pain was detected.

b Increased carotid intima-media thickness.

c Maternal measles during pregnancy.

d Ossification of the anterior spinal ligament.

e Renal artery stenosis with intimal proliferation.

*Termination.

Clinical characteristics of ARHR2 include short stature, leg deformities, recurrent fractures, bone pain, and muscle pain (2). Conventional treatment consists of phosphate and calcitriol replacement to normalize the serum ALP and counter FGF23-mediated 1α-hydroxylase suppression (1, 4, 6, 8–10). Bisphosphonates, synthetic analogues of PPi, have been used to suppress arterial calcification in GACI (2, 17), but no studies have reported a beneficial effect in ARHR2 patients. Burosumab, a monoclonal antibody against FGF23, was once considered a potential therapeutic strategy for correcting ENPP1 deficiency. However, the use of burosumab in ARHR2 patients is currently not recommended since it is not approved for this condition, and there exist concerns about worsening of ectopic calcification (18, 19). ENPP1 replacement therapy, currently being developed in animal models, may be a future treatment for ARHR2 (13, 20).

Although no evidence of arterial calcification was observed in this case, loss-of-function variants in *ENPP1* are also known to be associated with GACI (OMIM 208000), a life-threatening disease characterized by calcification of medium and large arteries (2). ENPP1 regulates mineralization by suppressing hydroxyapatite crystal deposition *via* hydrolysis of adenosine triphosphate into adenosine monophosphate and PPi (20). Therefore, ENPP1-deficiency results in pathological calcification and over-mineralization in vessels and soft tissues, resulting in GACI. Manifestations of GACI usually appear in early infancy, but often occur as early as *in utero*. GACI causes respiratory distress, cyanosis, and heart failure, and has a fatal course in the first 6 months of life in about half of patients (2, 21). ARHR2, which shares the same genetic change in *ENPP1* with GACI, usually manifests later in life (4, 21, 22). In one consanguineous family with a homozygous c.797G>T nonsynonymous sequence variant in *ENPP1*, the father (Case 13, **Table 2**) had rickets but no history of GACI, whereas his son (Case 14, **Table 2**) presented with GACI in the first week of life and later exhibited hypophosphatemia (1). Some studies have suggested that an inverse correlation between the degree of hypophosphatemia of ARHR2 and aberrant calcification of GACI might exist, indicating that low phosphate levels may protect the ENPP1-deficient patients from pathologic arterial calcification (3, 9, 17). Based on previous studies, it is currently believed that GACI and ARHR2 are on a spectrum of disease, although it is still unclear what determines the phenotype and when it occurs (2, 22).

In addition to ARHR2 and GACI, patients with a pathogenic variant in *ENPP1* gene can exhibit hearing loss, OPLL, PXE, and thrombocytopenia, hypoglycemia, neurologic or hepatic manifestations (4–6, 11, 23, 24). Although hearing loss is often observed in patients with X-linked hypophosphatemic rickets, it can also occur at an early age in patients with pathogenic variants in *ENPP1*, due to inner ear artery calcification or impaired inner ear development (23). Regular hearing examinations are required until adolescence in patients with *ENPP1*-related disorders, as the age of onset for hearing deficits ranges from a week after birth to 12 years of age (Cases 4, 5, 14, 16, and 17, **Table 2**) (1, 5, 6, 8, 23). OPLL also occurs in patients with *ENPP1* mutations (Cases 3, 5, and 15, **Table 2**) (4, 8, 10). A radiologic evaluation should be performed before conducting surgery in ARHR2 patients, since careful endotracheal intubation is needed to avoid hyperextension in cervical OPLL (25). As ENPP1 produces PPi, a potent endogenous inhibitor of hydroxyapatite crystal formation, it has been reported that certain ENPP1-deficient patients develop PXE, a disorder characterized by ectopic mineralization of the matrix in the skin, eyes, and cardiovascular system (5, 26). Reduced concentration of PPi is important in pathogenesis of both GACI and PXE (26), although it is not evaluated in this case report. To date, there have been no reports of PXE in ARHR2 patients until now (**Table 2**). However, monitoring patients with *ENPP1* pathogenic variants for cutaneous or ophthalmologic PXE lesions is recommended, considering the common genetic variation in PXE and ARHR2 (5).

The mother of the proband showed normal bone mineral density despite of leg pain and low TRP, and the father and the sister did not complain of any bone pain or neurological symptoms. None of the family members suffered from fractures. The phenotypes of heterozygous carriers of *ENPP1* pathogenic variants have not been well studied, but some researchers have suggested that mild hypophosphatemia, early-onset osteoporosis (12), or ossification/hyperostosis of the spinal ligament (27) could be associated with *ENPP1* haploinsufficiency. Although family members with heterozygous *ENPP1* variant in our case did not exhibit any of the aforementioned symptoms, periodic follow-up examinations are advised to detect possible manifestations later in life.

In conclusion, we report a rare case of ARHR2 caused by bi-allelic inactivating variants in *ENPP1* gene, diagnosed through NGS panel testing. The clinical phenotype was similar to that observed in previously reported cases of *ENPP1* variants. Since patients with ENPP1-deficiency can manifest various phenotypes including GACI, hearing loss, OPLL, and PXE, systematic evaluation should be mandatory for patients diagnosed with ARHR2.

## Data Availability Statement

The original contributions presented in the study are included in the article/**Supplementary Material**. Further inquiries can be directed to the corresponding author.

## Ethics Statement

The studies involving human participants were reviewed and approved by Institutional Review Board of the Seoul National University Hospital (No. 2107-157-1237). Written informed consent to participate in this study was provided by the participants’ legal guardian/next of kin. Written informed consent was obtained from the minor(s)’ legal guardian/next of kin for the publication of any potentially identifiable images or data included in this article.

## Author Contributions

YC, CHS, YAL, and YJL conceptualize the article. YC and MJK analyzed and interpreted the data. YC drafted the body of the manuscript. YAL, CHS, MJK, and YJL critically reviewed the publication. All authors contributed to the article and approved the submitted version.

## Funding

This research was supported by the Diagnosis Support Programs for Rare Diseases funded by the Korea Disease Control and Prevention Agency.

## Conflict of Interest

The authors declare that the research was conducted in the absence of any commercial or financial relationships that could be construed as a potential conflict of interest.

## Publisher’s Note

All claims expressed in this article are solely those of the authors and do not necessarily represent those of their affiliated organizations, or those of the publisher, the editors and the reviewers. Any product that may be evaluated in this article, or claim that may be made by its manufacturer, is not guaranteed or endorsed by the publisher.
