# Supplementary material for: Case Report and Review of Literature: Autosomal Recessive Hypophosphatemic Rickets Type 2 Caused by a Pathogenic Variant in ENPP1 Gene
Source: Front Endocrinol (Lausanne). 2022 Jul 29;13:911672. doi: 10.3389/fendo.2022.911672 (PMC9374118; doi:10.3389/fendo.2022.911672)
Supplement: Supplementary file 1 [file Table_1.docx]

# Supplementary Methods

Genomic DNA was extracted from the peripheral blood of the patient. The exon regions of the genome were targeted using the Agilent SureSelectXT Human All Exon 50 Mb kit. The Illumina HiSeq sequencing system with 100 bp paired-end reads was used to sequence targeted regions. The resulting DNA sequences were mapped to and compared with the UCSC GRCh37/hg19 human reference genome assembly. The mean depth of coverage was 152x, and 99.4% of the target bases had at least 10x coverage. The clinical significance of the sequence variant was classified according to the recent guidelines of American College of Medical Genetics and Genomics.

Supplementary Table 1. Twenty-one genes associated with rickets or calcium/phosphate metabolism disorders were included in the molecular panel.

| Genes |
| --- |
| *AIRE, AP2S1, CASR, CLCN5, CLDN16, CYP27B1, CYP2R1, DMP1, ENPP1, FAM111A, FAM20C, FGF23, GATA3, GCM2, GNA11, PHEX, PTH, SLC34A3, TBCE, TRPM6, VDR* |
